# Clinicopathological observations of colorectal serrated lesions associated with invasive carcinoma and high-grade intraepithelial neoplasm

**DOI:** 10.3892/etm.2013.1270

**Published:** 2013-08-23

**Authors:** SHENG XU, LUPING WANG, GUANGZHI YANG, LIN LI, JIN WANG, CHUNWEI XU, CHANG GE

**Affiliations:** Department of Pathology, General Hospital of Beijing PLA Military Region, Beijing 100700, P.R. China

**Keywords:** colorectal serrated lesion, invasive adenocarcinoma, immunohistochemistry, intraepithelial neoplasm

## Abstract

The aim of this study was to investigate the clinicopathological characteristics of colorectal serrated lesions associated with invasive carcinoma and high-grade intraepithelial neoplasm (HIN), as well as to determine the immunohistochemical expression of MutL homolog 1 (MLH1), MutS homolog 2 (MSH2), K-ras and O^6^-methylguanine-DNA methyltransferase (MGMT). A total of 5,347 cases diagnosed with colorectal polyp or adenoma were included in this study from October 2002 to September 2009. A total of 16 cases of colorectal serrated lesions associated with invasive carcinoma/HIN were screened. These comprised seven cases of traditional serrated adenoma (TSA) associated with invasive carcinoma and HIN, six cases of sessile serrated adenoma (SSA) associated with invasive carcinoma/HIN and three cases of hyperplastic polyp (HP) associated with invasive carcinoma/HIN. TSA associated with invasive carcinoma/HIN predominantly occurred in the rectum with a clearly serrated structure and ectopic crypts. High-grade dysplasia was observed in filiform TSA, which was more prone to carcinogenesis. SSA associated with invasive carcinoma/HIN mainly occurred in the ileocecal junction, with the SSA serrated glands closely located adjacent to the muscularis mucosa and the basal crypt expanded with inverted T- or L-shaped branches. HPs were observed in three cases in the cancer-adjacent tissues with invasive carcinoma, while a HP-SSA/TSA-carcinoma sequence was found in two cases. Immunohistochemistry showed that MGMT expression was significantly different in the serrated lesion tissues compared with that in cancer tissues (P=0.022), control cancer tissues (P=0.002) and normal colorectal epithelial tissues (P=0.003). TSA and SSA may progress to cancer or directly develop into invasive adenocarcinoma. Filiform TSA easily develops into HIN, followed by infiltration. HP may arise from the cancer-adjacent tissues of the invasive carcinoma, which are closely adjacent to the cancer tissues. Further research is needed to investigate the potential direct involvement of HP in carcinogenesis.

## Introduction

Colorectal carcinoma is a malignant tumor of the digestive tract that significantly threatens human health. It has been reported that ~60% of colorectal carcinomas arise from conventional adenomas, 35% from serrated adenomas and the remaining 5% from Lynch syndrome (1). In 1990, Longacre and Fenoglio-Preiser (2) described as ‘serrated adenomas’ a type of adenomas that are characterized by serrated architecture and dysplastic epithelium of the conventional adenomas. Serrated adenomas are considered to be preponderantly distributed in the cecum, rectum and sigmoid colon, with an intramucosal carcinoma incidence of 10%. Due to the advances in the investigation of serrated lesions, their clinical pathology and molecular genetics have been presented in detail in the WHO Classification of Tumors of the Digestive System (2010 version). A colorectal serrated lesion is defined in the new WHO Classification as a group of lesions characterized by serrated epithelial architecture including: hyperplastic polyp (HP), sessile serrated adenoma/polyp (SSA/P) and traditional serrated adenoma (TSA). Serrated adenocarcinoma is a recently described, distinct subtype of colorectal carcinoma (3,4). HP is the most common serrated lesion, which commonly occurs in distal colon and rectum with an incidence of 10–12.5%, which consists of 80–90% of all the serrated lesions (5). HP is characterized by the serrated profile of 1/3–1/2 of the upper glandular crypt and small cell nuclei that are regularly aligned close to the basement membrane (6,7). Moreover, the cells in HP are not characterized by atypia. TSA has a serrated characteristic structure and a morphological cytology that may be adequately used for the diagnosis of dysplasia (8). Ectopic crypt is a definitive TSA feature, which is a type of crypt foci that is not adjacent to the muscularis mucosa (9). SSA predominantly occurs in the proximal colon and it generally appears as a flat or sessile (broad-base) polyp that protrudes slightly. Under a microscope, the crypt of SSA is mainly characterized by a serrated morphology, which is closely adjacent to the muscularis mucosa. In addition, crypt expansion and deformity are observed. The bottom of the crypt broadens and appears as inverted T- or L-shaped branches (10). The complex gland of SSA may show dysplasia and pseudoinvasion (11).

HP is generally considered a type of benign lesion. Similarly to serrated precancerous lesions, SSA and TSA may progress to cancerous lesions through the serrated molecular genetic and epigenetic pathways, namely serrated neoplasia pathways. A recent study hypothesized that two serrated neoplasia pathways are involved in cancer development (1). The first is the sessile serrated neoplasia pathway with a mutated BRAF gene. The serrated lesion usually develops into serrated microvesicular HP (MVHP) and SSA/P accompanied by CpG island promoter methylation, which may lead to the silencing of some genes. When the human MutL homolog 1 (hMLH1) gene is silenced, cell atypia rapidly results in progression to SSA, followed by carcinogenesis. The second pathway, termed the traditional serrated pathway, is considered to be unrelated to the BRAF gene mutation; however, it is suggested to be involved in KRAS mutation. This pathway involves goblet-cell rich HP (GCHP) and TSA. Currently, there is limited knowledge concerning GCHP. Although it has been suggested that TSA is able to develop into cancer, the types of cancer that TSA develops into remain controversial. It has been reported that TSA has the potential to develop into microsatellite instability-low (MSI-L) colorectal cancer following methylation of the MTMG gene (1). However, further studies concerning the molecular mechanisms underlying the TSA-induced serrated neoplasia pathway are required.

Few cases with carcinogenesis of serrated lesions have been reported in countries other than China, and there is limited knowledge on the carcinogenesis of serrated lesions in China. In the present study, the medical files of 5,347 patients with polyps or adenomas were collected from five hospitals in Beijing and Hubei (China) during a 5-year period, along with 258 cases with serrated lesions that were screened, including 16 cases with serrated lesions associated with invasive carcinoma/HIN. The clinicopathological characteristics of the 16 cases were investigated and immunohistochemistry was performed. The aim was to aid further understanding of the carcinogenesis of the serrated lesions in order to provide evidence regarding the colorectal carcinogenesis pathways and the management of clinical prognosis of the patients.

## Subjects and methods

### Subjects

A total of 5,347 patients with polyps or adenomas were included in this study from October 2002 to September 2009. Patient diagnosis was performed following pathological examinations of the colon and rectum in the General Hospital of Beijing PLA Military Region (Beijing, China), 252 Hospital of Chinese PLA (Baoding, China), Navy General Hospital (Beijing, China), Julu County Hospital (Hubei, China) and Dongzhimen Hospital affiliated to Beijing University of Chinese Medicine (Beijing, China). All the polyp and adenoma sections were retrospectively reviewed. The sections were reviewed by three pathologists in 4–5 rounds using WHO Criteria to screen 258 cases of polyps and adenomas with serrated features (serrated lesions). Further histological diagnosis and classification was conducted in 258 cases with serrated lesions. A total of 16 cases with different types of serrated lesions were associated with invasive carcinoma/HIN, while 20 cases with colorectal adenocarcinoma served as controls. In addition, clinical and endoscopic data were collected for all the samples. All the subjects were informed of the study and provided written informed consent. The study was approved by the ethics committee of the hospital (The General Hospital Of Beijing PLA Military Region).

### Histological classification

According to the WHO Classification for the Tumours of the Digestive System (3,4), 258 cases of serrated lesions were classified as HP, SSA, TSA, mixed serrated polyp/adenoma and mixed serrated conventional adenoma. The histopathological changes of the 16 cases with serrated lesions associated with invasive carcinoma/HIN were then assessed. The lesions were grouped into the serrated lesion region, the corresponding cancer, carcinogenesis region and controls. The clinicopathological features of the serrated lesions were analyzed by combining clinical and endoscopic data.

### Immunohistochemical examination

Immunohistochemical staining was performed in 14/16 cases of cancerous serrated lesions, 20 cases of colorectal adenocarcinoma and 5 cases of normal colorectal mucosa based on primary antibodies against MLH1, MutS homolog 2 (MSH2), K-ras and O^6^-methylguanine-DNA methyltransferase (MGMT). All the specimens were fixed with 10% neutral buffered form-aldehyde, embedded in paraffin and cut into 4-*μ*m sections. Immunohistochemistry was performed using the MaxVision one-step immunohistochemical staining reagent kit following the manufacturer’s instructions (Beijing Zhongshan Golden Bridge Biotechnology Co., Ltd., Beijing, China). The primary antibodies used in the present study are shown in Table I.

Following immunohistochemical staining, K-ras was located on the cell membrane, while MLH1, MSH2 and MGMT were located on the cell nucleus. The percentage of positively immunostained cells was determined using a method previously described (12). The region with five clear well-structured positive cells was sampled from each section, and the percentage of positively stained cells (not including interstitial or non-tumor cells) in 100 cells was counted in each region under a microscope at high magnification. The mean percentage of positively stained cells was calculated based on 5 counts. Immunohistochemical staining was classified into 4 grades based on the percentage of positively stained cells: grade I, score 0; II, score 1; III, score 2; and IV, score 3 with <25, 26–50, 51–75 and 76–100% positively stained cells, respectively.

The presence of clear brown-yellow granules was evaluated as positive staining, and the intensity of immunohistochemical staining was classified into five grades: colorless as grade 0 (score 0); light yellow as grade I (score 1); yellow as grade II (score 2); brown yellow as grade III (score 3); and brown as grade IV (score 4). The multiplication of two types of scores in each section was defined as the final score of the expression intensity. Scores 0, 1–4, 6–8 and >8 were marked as (−), (+), (++) and (+++), respectively; while (−) to (+) was defined as a reduced expression or loss of expression.

### Statistical analysis

All the statistical analyses were performed using the statistical software SPSS 13.0 (SPSS, Inc., Chicago, IL, USA). Chi-square and Kruskal-Wallis H tests were used for statistical analyses. P<0.05 was considered to indicate a statistically significant difference.

## Results

### Histopathological observations

**Figure 1.**
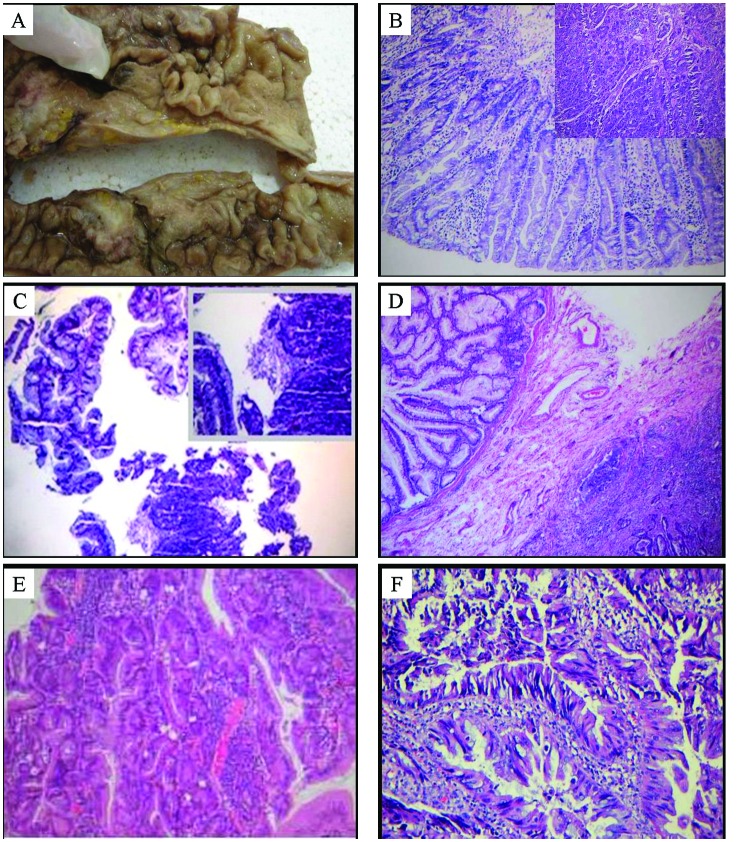
(A) General view of HP associated with low-differentiated adenocarcinoma. The black arrow indicates the tangent plane of colon cancer, and the red arrow indicates HP. (B) Microscopic observation of the HP shown in (A). The lesion conformed to GCHP and appeared as serrated changes. There were many goblet cells in the gland with the nucleus mildly stained and closely adjacent to the basement, without any atypia. The small figure in the right upper corner is a microscopic view of the moderately differentiated adenocarcinoma (Hematoxylin and eosin, magnification ×100). (C) Filiform TSA associated with carcinogenesis. The left upper half indicates the filiform serrated lesions, and the lower half and the right upper corner indicate the carcinogenesis area (Hematoxylin and eosin, magnification ×100). (D) SSA associated with moderately differentiated adenocarcinoma, and the crypt of the SSA exhibited an inverted L shape, which was closely adjacent to the mucosal muscularis. Arrows indicate invasive cancer tissues adjacent to the SSA (Hematoxylin and eosin, magnification ×100). (E) SSA associated with HIN. Glands exhibited marked atypia (Hematoxylin and eosin, magnification ×100). (F) SSA associated with SAC. Disorder of the serrated structures was observed in SAC, where cell nuclei were vacuole-like shaped and the cytoplasm was acidophilic (Hematoxylin and eosin, magnification ×200). HP, hyperplastic polyp; GCHP, goblet-cell rich HP; TSA, traditional serrated adenoma; SSA, sessile serrated adenoma; HIN, high-grade intraepithelial neoplasm; SAC, serrated adenocarcinoma.

The 16 cases with serrated lesions associated with invasive carcinoma/HIN showed the following characteristics when observed under a microscope: i) there were three cases of HP that appeared with serration along with a narrow and small basement membrane. The nucleus of the cells in the HP was small and regular, and was located close to the basement membrane. Differentiated and mature goblet cells along with the columnar cells were abundant in the HP without any evident atypism. The invasive carcinoma tissues from the three patients were closely adjacent to the HP including two cases with moderate- or low-differentiated adenocarcinoma (Fig. 1A and B) and one case with local serrated adenocarcinoma. HP was observed in the cancer-adjacent regions of the three cases with invasive adenocarcinoma, where a close association was detected. However, the polyp-cancer sequence was not fully clear. ii) There were seven cases of TSA associated with malignancy in which the crypt of the TSA appeared as an evident serration along with ectopic crypts. The serrated crypt was not adjacent to the mucosal muscularis and the epithelial cells showed an apparent atypism, which was characterized by different degrees of dysplasia and even HIN. The cell nuclei appeared to undergo rod- or vacuole-like changes and the cytoplasm of the cells was acidophilic. Out of the seven patients, five cases were classified as filiform serrated adenomas, a specific subtype of TSA characterized by long villiform extensions and serration. Epithelial cells in the TSA appeared as non-mucosal high-columnar-shaped cells with an acidophilic cytoplasm along with some atypism. Among the seven cases of TSA, two cases were associated with HIN (Fig. 1C), one with the sequence HP/TSA and cancerated tissues without any distinct infiltration observed, while four cases had cancerated tissues associated with infiltration. iii) There were six patients with SSA associated with malignancy (Fig. 1D and E). The crypt of the SSA with serrated changes was closely adjacent to the mucosal muscularis and was close to the cancer tissues. The crypt of the adenoma was markedly expanded and distorted, while it was broadened towards the basement membrane, appearing as inverted T- or L-shaped branches. The majority of the cell nuclei were elongated, appearing with a rod-like shape. Some cells on the middle and upper layers of the crypt showed atypia and the formation of pseudostratified layers, while certain other cells only showed abnormalities in the crypt structure without an apparent cell atypia. The 6 patients with SSA included one case associated with serrated adenocarcinoma (Fig. 1F), one associated with mucinous adenocarcinoma, three associated with common adenocarcinoma and one associated with HP/SSA and cancerated tissues. iv) Features of serrated adenocarcinomas were also observed. The 16 patients with serrated lesions were associated with invasive carcinoma/HIN and the cancer tissues from two additional cases had pathological features of serrated adenocarcinoma (Fig. 1F). In addition to the common characteristics of the cancer tissues, such as invasive growth and atypia of glandular cells, serrated adenocarcinoma was characterized by serrated cancer tissues, acidophilic cytoplasm, abundant mucus in peri-cancer tissues, rod- or vacuole-shaped cell nuclei and a relatively low percentage of karyoplasm.v) An HP-SSA-Adenocanceration sequence was observed in one case, and an HP-TSA-carcinogenesis sequence was observed in another case.

### Clinical features of the patients with colorectal polyps/adenomas

Out of the 5,347 patients with colorectal polyps/adenomas sampled, a total of 258 cases had serrated lesions and 16 cases had serrated lesions associated with invasive carcinoma/HIN, which consisted of 0.297% of the total number of patients with polyps or adenomas and 6.2% of the patients with serrated lesions, respectively. Among the 16 patients with serrated lesions associated with invasive carcinoma/HIN, seven cases had TSA associated with invasive carcinoma/HIN, which comprised 2.71% of the total number of patients with serrated lesions; six cases had SSA associated with invasive carcinoma/HIN, which comprised 2.33% of the total number of patients with serrated lesions; and three cases had HP associated with invasive carcinoma, which comprised 1.16% of the total number of patients with serrated lesions.

**Figure 2.**
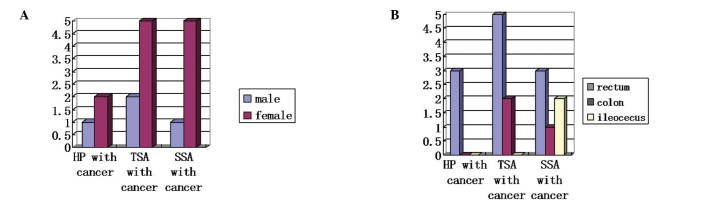
(A) Patient gender and (B) the sites of serrated lesions with carcinogenesis. HP, hyperplastic polyp; TSA, traditional serrated adenoma; SSA, sessile serrated adenoma.

The 16 patients with serrated lesions associated with invasive carcinoma/HIN included four males and 12 females; Two males and five females with TSA associated with invasive carcinoma/HIN, one male and five females with SSA associated with invasive carcinoma/HIN, and one male and two females with HP associated with invasive carcinoma. This indicated that the serrated lesions associated with invasive carcinoma/HIN predominantly occurred in females. All three cases of HP were associated with invasive carcinoma in the rectum. In the seven patients with TSA, the site of the associated invasive carcinoma/HIN was the rectum in five cases, the descending colon in one case and the ascending colon in one case. In the six cases of SSA, the associated invasive carcinoma/HIN was located in the rectum in three cases, in the ileocecal junction in two cases, and in the sigmoid colon in one case. The onset of HP associated with the development of invasive carcinoma occurred at the age of 44–48 years with a mean age of 46.3 years; the onset of TSA associated with invasive carcinoma occurred at the age of 38–77 years with a mean age of 63.1 years, while the onset of SSA associated with invasive carcinoma occurred at the age of 42–60 years with a mean age of 51 years (Table II and Fig. 2).

### Immunohistochemical observations

**Figure 3.**
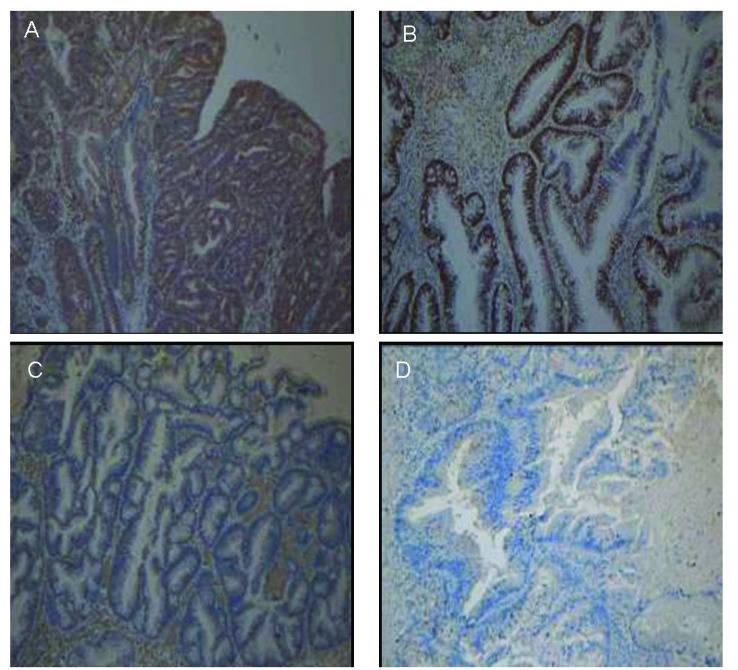
Positive expression of (A) K-ras and (B) MSH2 in serrated lesions, and negative expression of MGMT and MLH1 in (C) SSA and (D) SAC. MSH2, MutS homolog 2; MGMT, O^6^-methylguanine-DNA methyltransferase; MLH1, MutL homolog 1; SSA, sessile serrated adenoma; SAC, serrated adenocarcinoma.

Immunohistochemistry was performed to determine the 14 patients with serrated lesions associated with invasive carcinoma/HIN. The determination of MLH1, MSH2, K-ras and MGMT expression was conducted in the serrated lesions and their complicating cancer tissues (experimental group) in the 20 patients with common tubular adenocarcinoma and five cases with colorectal mucosal tissues (control group) as shown in Table III and Fig. 3. No significant differences were observed in the expression of K-ras, MSH2, MLH1 and MGMT in the cancer tissues from the patients in the experimental group compared with those in the control group (P=0.954, 0.809, 0.447 and 0.500, respectively). Furthermore, the expression of K-ras, MSH2 and MLH1 in the serrated lesions from the experimental group was not significantly different compared with that of the cancer tissues from the control group (P=0.954, 0.500 and 0.861, respectively); however, a significant difference was detected in MGMT expression (P=0.002). The expression of K-ras, MSH2 and MLH1 in the serrated lesions from the experimental group was not observed to be significantly different compared with that of the cancer tissues from the experimental group (P<1.000, 0.403 and 0.357, respectively), while a significant difference in the expression of MGMT was found between the groups (P=0.0022). The expression of K-ras, MSH2 and MLH1 in the serrated lesions from the experimental group was not significantly different compared with that of the normal colorectal mucosal tissues (P=0.384, 0.145 and 0.190, respectively), while a significant difference was observed between MGMT expression in the two groups (P=0.003). Furthermore, no significant differences in the expression of K-ras, MSH2, MLH1 and MGMT among the three types of serrated lesions including HP, SSA and TSA were observed (P<0.736, 0.969, 0.255 and 0.373, respectively). MGMT expression was reduced in the serrated lesions compared with the expression in the control group, while no significant difference was observed in the MGMT expression among the three types of serrated lesions. A reduced expression of MLH1 was detected in the four patients with serrated lesions, including two cases with reduced MLH1 expression in the complicating cancer tissues.

## Discussion

SSA and TSA have been reported to be neoplastic polyps, which are precancerous lesions, while HP is a type of non-neoplastic lesion that has no malignant potential (13). However, polyps (including HP) with a serrated architecture have been reported to possess a malignant potential (14). A HP with a diameter of ≥10 mm has been shown to increase the risk of development of colorectal carcinoma since the molecular background of these polyps is similar to that of the corresponding subtypes of colorectal carcinoma (15). In the present study, two patients with HP associated with SSA/TSA and cancerous tissues were identified; their histological transition process was observed and immunohistochemical analysis revealed similar results. MGMT expression was reduced in HP and SSA/TSA, while the normal expression of MGMT was detected in the correlated cancer tissue, suggesting that HP may be transformed into TSA or SSA, followed by carcinogenesis. Our findings suggest that HP has a malignant potential, and that follow-up should be strictly practiced in patients with large HPs. In addition, three cases of invasive adenocarcinoma had structures of HP in their cancer-adjacent region. Immunohistochemical investigation showed a positive expression of MGMT in cancer tissues with a reduced expression of MGMT in HPs. Another case had negative expression of MGMT in cancer tissues and HP. Due to the limited number of the cases included in the present study, further studies are needed to investigate the expression of MGMT in cancer tissues and HPs.

SSA and TSA are precancerous lesions of colorectal carcinoma, which may be followed by cancer development. It has been indicated that 5.3% of serrated adenomas and 2.2% of conventional adenomas developed into cancer. Serrated adenomas with evident dysplasia are suggested to have a higher malignant tendency compared with conventional adenomas (16). However, the malignant potential of serrated adenomas is considered to be lower compared with that of conventional adenomas (3.2 vs. 9.3%) (17). Serrated adenomas have a clear malignant potential; however, there are fewer cases of serrated adenomas associated with high-grade intraepithelial neoplasm and invasive adenocarcinoma than of cases with conventional adenomas (18). The progression of malignancy of serrated lesions is faster compared with that of conventional adenomas (19). Based on the example of the sessile serrated pathway, the time frame of development from HP to SSA is unknown; however, SSA rapidly develops into cancer, where <10 years are required for the detection of a clear dysplasia. It has been estimated that ~5% of SSAs develop into cancer within 20 years, while only 2.2% of conventional adenomas develop into cancer (20). Currently, there are two major serrated neoplasia pathways. The sessile serrated adenoma pathway usually has high microsatellite instability (MSI-H). The mechanism of carcinogenesis is mainly explained by BRAF mutation-induced methylation of the mismatch repair gene MLH1 and a high level of CpG island methylation, resulting in different degrees of dysplasia and finally carcinogenesis, which is known as the sessile serrated pathway. While the traditional serrated adenoma-induced colorectal carcinoma usually has a low microsatellite instability (MSI-L) and its mechanism of carcinogenesis is mainly K-ras mutation-induced methylation of the MGMT DNA repair gene and a low level of CpG island methylation, which is known as the traditional serrated pathway (1,21).

The present study determined the expression of the four antibodies K-ras, MSH2, MLH1 and MGMT in serrated lesions associated with invasive cancer/HIN, while common colorectal carcinoma and normal colorectal mucosal tissues served as controls. MLH1 and MSH2 are mismatching repair genes, which are associated with MSI. Immunohistochemical staining of SSA with MSI-H and SSA-related cancer tissues indicated a significant loss of MLH1 expression in the SSA-associated high-grade dysplasia region or intra-mucosal carcinoma regions (22). The expression of the mismatch repair gene was lost in SSA adjacent to the cancer tissues or dysplasia regions (23). MGMT is a DNA repair gene, and its methylation and loss of expression have been detected in sessile and traditional serrated pathways, particularly in traditional serrated pathway-associated lesions, such as TSA (24). TSA with high-grade dysplasia has been shown to have a significantly higher frequency of K-ras mutation and MGMT methylation (25). In the present study, immunohistochemical analysis showed a positive expression of K-ras and MSH2 in serrated lesions, cancer tissues and controls, and no significant difference was observed among them (Fig. 3A and B). MGMT expression was significantly lower in serrated lesion tissues compared with that of complicating cancer tissues (P=0.022), control cancer tissues (P=0.002) and normal colorectal mucosal tissues (P=0.003). However, MGMT expression in cancer tissues from patients in the experimental group was not significantly different from that of the controls (P=0.500). Although no significant differences in the MLH1 expression were identified among the groups, a reduced expression of MLH1 was detected in one case with SSA, one case with HP and the complicating cancer tissues (serrated adenocarcinoma). Furthermore, a reduction in the MLH1 expression was also observed in one case with TSA and one case with HP, while no changes in MLH1 expression were identified in the complicating cancer tissues. Immunohistochemical analysis of 14 patients with serrated lesions associated with invasive cancer/HIN showed different expression levels of MGMT in the majority of the serrated lesions and their complicating cancer tissues, while similar expression was detected in cancer tissues from patients in the experimental and the control groups. Consistent MGMT and MLH1 expression was observed in serrated lesions and their complicating cancer tissues from certain patients, suggesting that the mechanism underlying the carcinogenesis of serrated lesions is more complex than expected. Furthermore, serrated lesions are suggested to transform into common or serrated adenocarcinomas.

It has been reported that ~17% of the serrated adenocarcinomas are adjacent to the SSA with different degrees of dysplasia (26). According to WHO and Makinen’s criteria (21), serrated adenocarcinoma is diagnosed based on the following features: i) crypt epithelium with serration; ii) acidophilic or neutrophilic cytoplasm; iii) abundant cytoplasm with a clear vacuole-like nuclei; iv) no necrosis on the tumor surface, or an area of necrosis of <10% of the cell surface area; v) presence of the mucus components; and vi) spherical cells with a long mastoid rod-like process in mucins of the tumors. In the present study, in 2/16 cases, the cancerated tissues from the colorectal serrated lesions associated with invasive carcinoma/HIN conformed to the diagnosis of serrated adenocarcinoma, including one case of sessile serrated adenoma associated with serrated adenocarcinoma and one case of HP associated with the components of serrated adenocarcinoma. Immunohistochemical analysis showed that the negative expression of MLH1 in sessile serrated adenomas was associated with serrated adenocarcinoma. Our findings showed that not all the cancerated serrated lesions were serrated adenocarcinomas; however, some might develop into tubular adenocarcinomas. Serrated adenocarcinoma is defined as a subtype of colorectal carcinoma in the WHO Classification of Tumors of the Digestive System (2010 version). However, further studies are required for the investigation of the clinicopathological significance of serrated adenocarcinomas.

The present study demonstrated that TSA or HP associated with invasive carcinoma/HIN occurred predominantly in the rectum, while SSA associated with invasive carcinoma/HIN mainly occurred in the ileocecal junction (Fig. 2B). This was in agreement with the majority of previous studies (18). In addition, the age of the patients at the onset of TSA and HP associated with invasive carcinoma/HIN in this study was similar to the age of onset in previous studies. Also, the age of the patients was lower at the onset of HP associated with invasive carcinoma/HIN compared with the age of onset of TSA-associated with invasive carcinoma/HIN. The 16 cases of colorectal serrated lesions associated with invasive carcinoma/HIN were screened among 5,347 patients with polyps or adenomas, and included 4 males and 12 females. The male:female ratios of the patients with TSA, SSA and HP associated with invasive carcinoma/HIN were 1:2, 1:4 and 1:4, respectively. This indicated that the number of female patients was significantly greater compared with that of males (fig. 2A). This is significantly different the findings of previous studies, which reported that more male than female patients presented with serrated lesions (27).

In conclusion, TSA and SSA/P may develop into invasive adenocarcinoma directly, and filiform serrated adenoma (FSA) tend to evolve into HIN easily. HP may be adjacent to invasive adenocarcinoma tissues, however, further observation is required to investigate whether it may directly develop into cancer. The results of immunohistochemistry showed that there was no expression of MGMT in serrated lesions and 2 cases of SAC. There was no expression of MLH1 in SSA/P. The results indicated that there were different molecular pathways in different types of serrated lesions.

## Figures and Tables

**Table I.** Origin, concentration and expression site of antibodies.

Antibody	Clone number	Manufacturer	Work concentration	Expression site
MLH1	G168–728	Beijing Zhongshan Golden Bridge Biotechnology Co., Ltd.	Ready-to-use	Cytoblast
MSH2	G219–1129	Beijing Zhongshan Golden Bridge Biotechnology Co., Ltd.	Ready-to-use	Cytoblast
K-ras	Polyclonal	Beijing Zhongshan Golden Bridge Biotechnology Co., Ltd.	Ready-to-use	Cytoplasm
MGMT	MT23.2	Beijing Zhongshan Golden Bridge Biotechnology Co., Ltd.	Ready-to-use	Cytoblast

MLH1, MutL homolog 1; MSH2, MutS homolog 2; MGMT, O^6^-methylguanine-DNA methyltransferase.

**Table II.** Clinicopathological features of 16 patients with serrated lesions associated with invasive cancer/HIN.

Case no.	Age (years)	Gender	Site of lesion	Pathological diagnosis and classification	Presence of infiltration
1	48	Male	Rectum	HP associated with moderate- or high-differentiated adenocarcinoma (some are SAC)	Yes
2	47	Female	Rectum	GCHP in the low-differentiated adenocarcinoma-adjacent region	Yes
3	44	Female	Rectum	Moderate-differentiated adenocarcinoma, HP located on the cancer-adjacent region	Yes
4	68	Female	Rectum	TSA associated with moderate-differentiated adenocarcinoma	Yes
5	53	Female	Rectum	HP and TSA associated with HIN	Yes
6	38	Female	Descending colon	Filiform TSA associated with moderate-differentiated adenocarcinoma	Yes
7	77	Female	Ascending colon	Filiform TSA associated with HIN	Not found
8	75	Female	Rectum	Filiform TSA associated with HIN	Not found
9	59	Male	Sigmoid colon/rectum	Filiform TSA of the sigmoid colon associated with rectal adenocarcinoma	Yes
10	72	Male	Rectum/sigmoid colon	HP of the sigmoid colon, and filiform TSA associated with invasive cancer in the rectum	Yes
11	52	Male	Rectum	SSA associated with carcinogenesis, and some developed mucosal carcinoma	Yes
12	47	Female	Ileocecal junction	SSA associated with SAC	Local
13	56	Female	Ileocecal junction	SSA associated with HIN	Local
14	42	Female	Rectum/sigmoid colon	SSA of the sigmoid colon associated with HIN	Not found
15	49	Female	Rectum	SSA associated with moderate- or high-differentiated adenocarcinoma	Yes
16	60	Female	Rectum	SSA associated with HIN	Not found

TSA, traditional serrated adenoma; SSA, sessile serrated adenoma; HP, hyperplastic polyp; GCHP, Goblet-cell rich hyperplastic polyp; SAC, serrated adenocarcinoma; HIN, high-grade intraepithelial neoplasia.

**Table III.** Immunohistochemical detection of serrated lesions associated with invasive cancer/HIN.

Group	K-ras			MSH2			MLH1			MGMT			
	−/+	++ to +++	Positive rate (%)	−/+	++ to +++	Positive rate (%)	−/+	++ to +++	Positive rate (%)	−/+	++ to +++	Positive rate (%)	n
Experimental													
Carcinogenesis	2	12	85.7	3	11	84.6	2	12	85.7	5	9	64.3	14
Serrated lesions													
HP	0	3	100	1	2	66.7	2	1	33.3	2	1	33.3	3
SSA	1	5	83.3	2	4	66.7	1	5	83.3	4	2	33.3	6
TSA	1	4	80	2	3	60	1	4	80	5	0	0	5
Control													
Carcinogenesis	3	17	85	5	15	75	5	15	75	5	15	75	20
Normal	0	5	100	3	2	40	0	5	100	0	5	100	5

MSH2, MutS homolog 2; MLH1, MutL homolog 1; MGMT, O^6^-methylguanine-DNA methyltransferase; HP, hyperplastic polyp; SSA, sessile serrated adenoma; TSA, traditional serrated adenoma.
